# Supplementary material for: Probing the functional constraints of influenza A virus NEP by deep mutational scanning
Source: Cell Rep. Author manuscript; Available in PMC 2025 Feb 16. (PMC11830162; doi:10.1016/j.celrep.2024.115196)
Supplement: 1 [file NIHMS2052773-supplement-1.pdf]

Cell Reports, Volume *44*

Supplemental information

Probing the functional constraints of influenza A virus NEP by deep mutational scanning

Qi Wen Teo, Yiquan Wang, Huibin Lv, Michael S. Oade, Kevin J. Mao, Timothy J.C. Tan, Yang Wei Huan, Joel Rivera-Cardona, Evan K. Shao, Danbi Choi, Chaoyang Wang, Zahra Tavakoli Dargani, Christopher B. Brooke, Aartjan J.W. te Velthuis, and Nicholas C. Wu

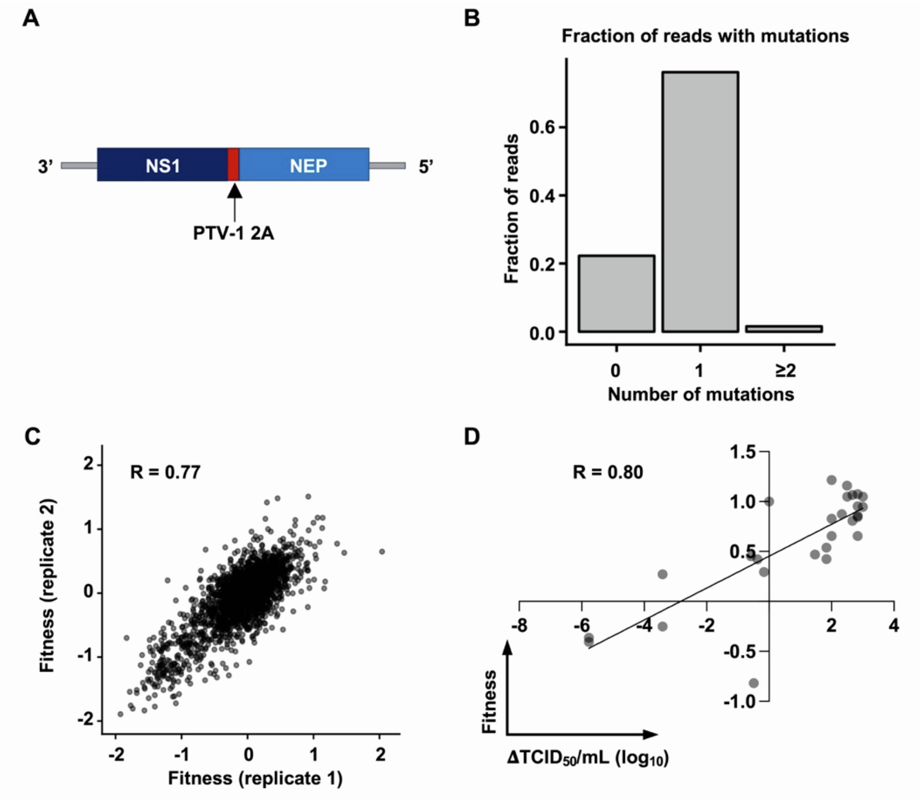

Supplementary Figure 1. Schematic representation of the NS chimeric gene construct, **Related to Figure 1**. **(A)** A NS-split construct was used for virus rescue experiments [S1]. The NS1 and NEP coding sequences were separated by the porcine teschovirus-1 2A autoprotease cleavage site (PTV-1 2A). **(B)** Distribution of amino acid mutations per clone in the NEP mutant library was analyzed by next-generation sequencing and is shown as a bar plot. **(C)** Correlation of fitness values of individual mutations between two independent biological replicates is shown as a scatterplot. **(D)** Correlation between fitness values of individual mutations and their titer in virus rescue experiments is shown as a scatterplot. Here, virus rescue titer was normalized by subtracting the virus rescue titer of WT from that of each mutant, since the data were from two virus rescue experiments with two different batches of cells (**Figure 2A-B**).

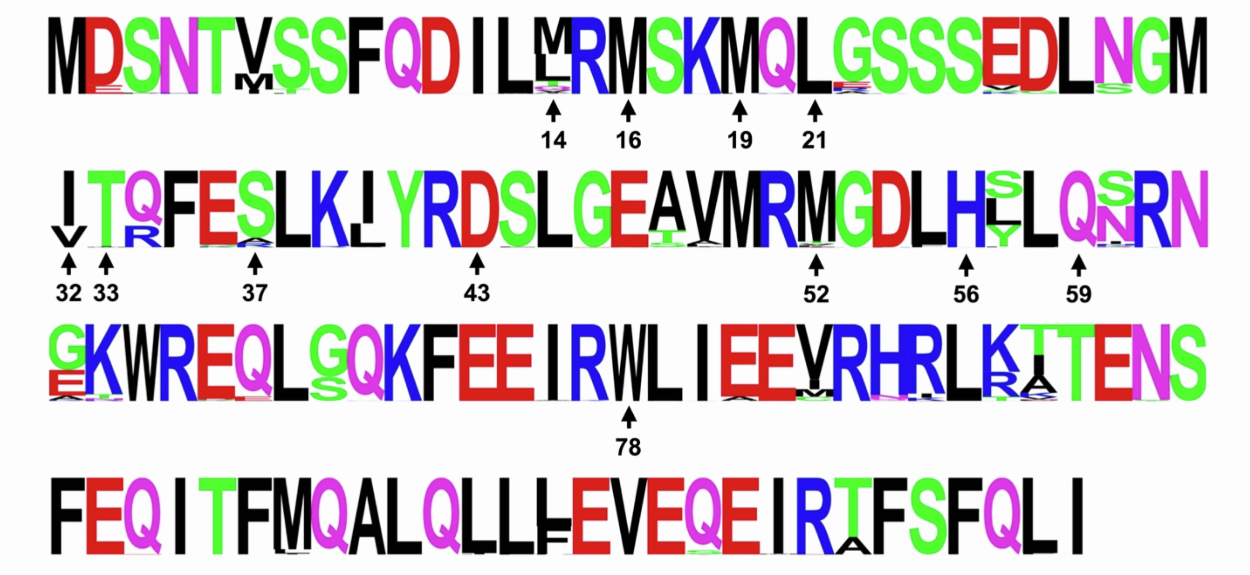

Supplementary Figure 2. Sequence conservation of influenza NEP, Related to Figure 1. The natural variants of NEP residues are represented by a sequence logo. The analysis includes sequences with the full length of NEP (121 amino acid residues) from all influenza strains on Influenza Virus Database [S2]. The amino acid residues mentioned in the main text are indicated.

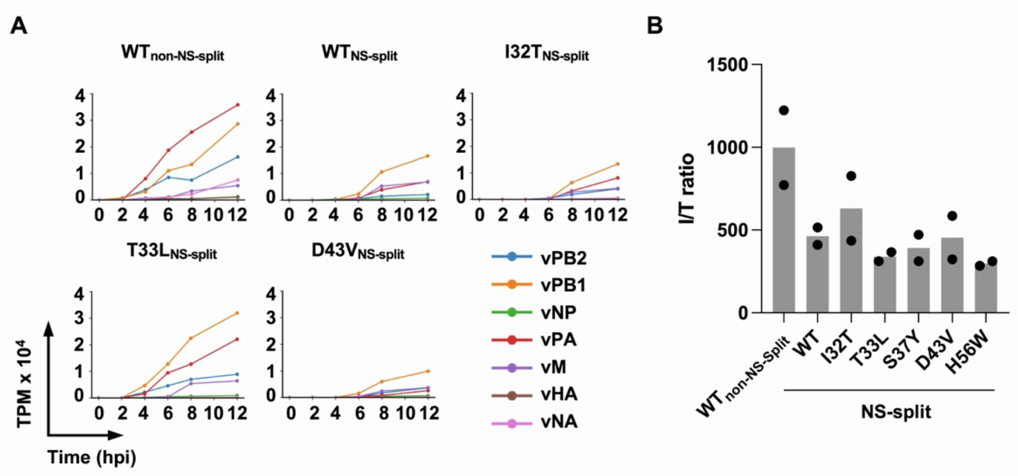

Supplementary Figure 3. Analysis of defective interfering particles (DIPs), Related to Figure 2. (A) Frequency of DIPs across viral gene segments in infected cells was analyzed by total RNA sequencing. Each data point represents the mean Transcripts Per Million (TPM) of three replicates. **(B)** Ratio of infectivity titer (I) to total titer (T). Non-infectious particles were measured using the hemagglutinin assay, while infectious particles were quantified using the $TCID_{50}$. The I/T ratio was computed by dividing the infectivity titer (Log_{10} $TCID_{50}$/50 μL) to the total titer (HA/50 μL) [S3]. Each bar represents the mean of two independent virus stocks. The differences between different mutants and WT are not statistical significance based on one-way ANOVA with Turkey's honestly significant difference post hoc test.

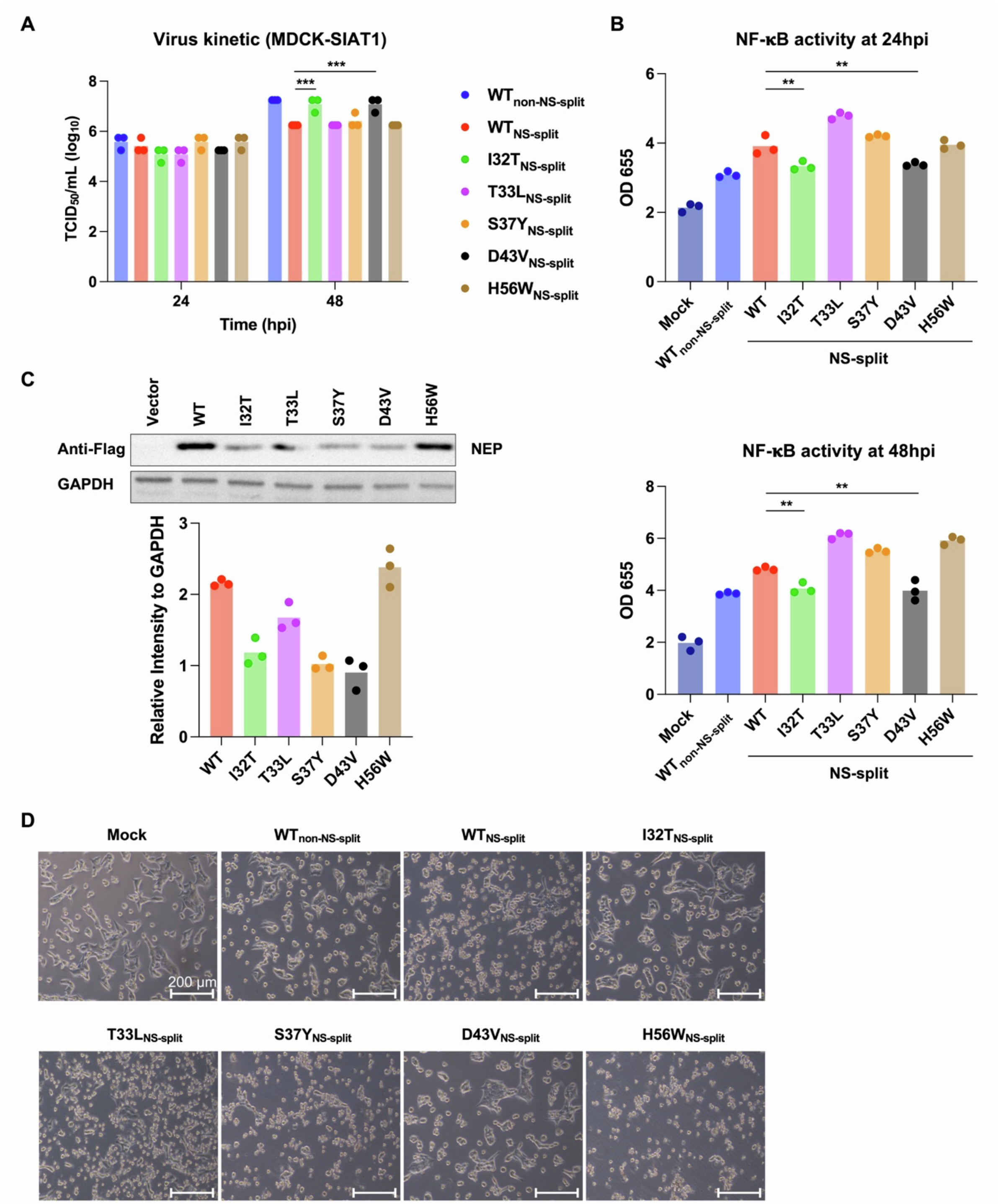

Supplementary Figure 4. Impact of mutations in N-terminal domain of NEP on cellular responses, Related to Figure 4. (A) Growth curves of wild-type (WT) and NEP mutant viruses in MDCK-SIAT1 cells. MDCK-SIAT1 cells were infected at an MOI of 0.001 with the indicated virus. Infectious particles in the supernatant were harvested at the indicated timepoints and quantified

by the $TCID_{50}$ assay. Each bar represents the mean of three independent biological replicates. Statistical significance was analyzed by one-way ANOVA with Turkey's honestly significant difference post hoc test: ***$P < 0.001$. **(B)** Activation of NF-κB activity was measured in mock- and IAV-infected A549 cells expressing dual reporters for NF-κB and IRF activities. Each bar represents the mean of three independent biological replicates. Statistical significance was analyzed by one-way ANOVA with Turkey's honestly significant difference post hoc test: **$P < 0.01$. **(C)** Western blot analysis of NEP protein expression. Flag-tagged NEP was transiently expressed in HEK293T cells and detected by an anti-Flag antibody. NEP protein expression was quantified by ImageJ image processing program [S4]. Glyceraldehyde 3-phosphate dehydrogenase (GAPDH) was used as an internal control to normalize data. Each bar represents the mean of three independent biological replicates. **(D)** A549 cells were infected by the indicated recombinant viruses at an MOI of 0.1. Cell death was assessed using phase contrast microscopy at 24 hpi. Scale bar: 200 μm.

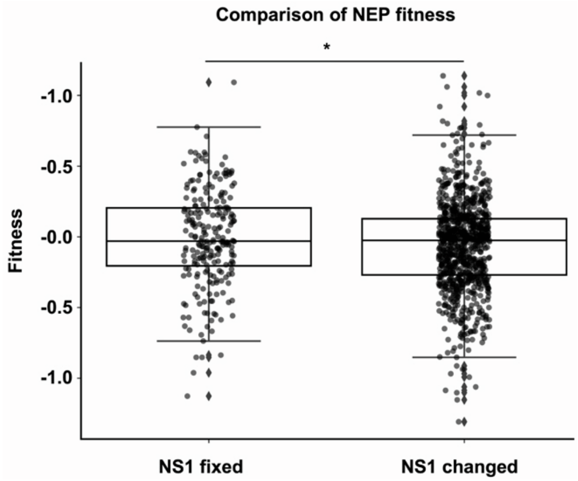

Supplementary Figure 5. Comparison of NEP fitness, Related to Figure 1. The fitness values between NEP mutations that would have altered the NS1 amino acid sequence and those would not in the context of $WT_{non\text{-}NS\text{-}split}$ were compared using two-tailed Student's unpaired t-test: $^*P < 0.05$.

SUPPLEMENTARY REFERENCES

S1. Basler, C.F., Reid, A.H., Dybing, J.K., Janczewski, T.A., Fanning, T.G., Zheng, H., Salvatore, M., Perdue, M.L., Swayne, D.E., and García-Sastre, A. (2001). Sequence of the 1918 pandemic influenza virus nonstructural gene (NS) segment and characterization of recombinant viruses bearing the 1918 NS genes. PNAS *98*, 2746-2751.

S2. Bao, Y., Bolotov, P., Dernovoy, D., Kiryutin, B., Zaslavsky, L., Tatusova, T., Ostell, J., and Lipman, D. (2008). The influenza virus resource at the National Center for Biotechnology Information. J Virol *82*, 596-601.

S3. Xue, J., Chambers, B.S., Hensley, S.E., and López, C.B. (2016). Propagation and characterization of influenza virus stocks that lack high levels of defective viral genomes and hemagglutinin mutations. Front Microbiol *7*, 326.

S4. Schneider, C.A., Rasband, W.S., and Eliceiri, K.W. (2012). NIH Image to ImageJ: 25 years of image analysis. Nat Methods *9*, 671-5. 10.1038/nmeth.2089.
